# Role of DNA Methylation in the Resistance to Therapy in Solid Tumors

**DOI:** 10.3389/fonc.2020.01152

**Published:** 2020-08-07

**Authors:** Susana Romero-Garcia, Heriberto Prado-Garcia, Angeles Carlos-Reyes

**Affiliations:** Department of Chronic-Degenerative Diseases, National Institute of Respiratory Diseases “Ismael Cosío Villegas”, Mexico City, Mexico

**Keywords:** DNA methylation, tumor suppressor genes, oncogenes, DNMTs, therapeutic targets, biomarkers, solid tumors, chemotherapy

## Abstract

Despite the recent advances in chemotherapeutic treatments against cancer, some types of highly aggressive and invasive cancer develop drug resistance against conventional therapies, which continues to be a major problem in the fight against cancer. In recent years, studies of alterations of DNA methylome have given us a better understanding of the role of DNA methylation in the development of tumors. DNA methylation (DNAm) is an epigenetic change that promotes the covalent transfer of methyl groups to DNA. This process suppresses gene expression through the modulation of the transcription machinery access to the chromatin or through the recruitment of methyl binding proteins. DNAm is regulated mainly by DNA methyltransferases. Aberrant DNAm contributes to tumor progression, metastasis, and resistance to current anti-tumoral therapies. Aberrant DNAm may occur through hypermethylation in the promoter regions of tumor suppressor genes, which leads to their silencing, while hypomethylation in the promoter regions of oncogenes can activate them. In this review, we discuss the impact of dysregulated methylation in certain genes, which impact signaling pathways associated with apoptosis avoidance, metastasis, and resistance to therapy. The analysis of methylome has revealed patterns of global methylation, which regulate important signaling pathways involved in therapy resistance in different cancer types, such as breast, colon, and lung cancer, among other solid tumors. This analysis has provided gene-expression signatures of methylated region-specific DNA that can be used to predict the treatment outcome in response to anti-cancer therapy. Additionally, changes in cancer methylome have been associated with the acquisition of drug resistance. We also review treatments with demethylating agents that, in combination with standard therapies, seem to be encouraging, as tumors that are in early stages can be successfully treated. On the other hand, tumors that are in advanced stages can be treated with these combination schemes, which could sensitize tumor cells that are resistant to the therapy. We propose that rational strategies, which combine specific demethylating agents with conventional treatment, may improve overall survival in cancer patients.

## Introduction

During carcinogenesis, genetic and epigenetic alterations lead to dysregulated expression of genes associated with cellular pathways that regulate processes such as cell proliferation, cell differentiation, cell death, and cell cycle, among others. Epigenetic alterations that include DNA methylation (DNAm), histone modifications, aberrant expression of microRNAs (miRNAs), and long non-coding RNA (lncRNA) are common in several types of cancer. These epigenetic changes are hereditary, transient, and reversible and do not cause modification in the DNA sequence (1). Cancer can be treated by resection, chemotherapeutic agents, radiation, and immunotherapy, among others, as well as any combination of the aforementioned therapies. However, the 5-year survival rate remains low in many solid tumors due to tumor intrinsic or acquired resistance (2).

DNAm is a pivotal mechanism in normal cell development, which plays an important role in the regulation of gene expression, as well as chromatin stability, genetic imprinting, X-chromosome inactivation, the suppression of repetitive element transcription, and transposition. In mammals, DNAm involves the covalent transfer of methyl groups (-CH_3_) from S-adenosyl-1-methionine (SAM) to cytosine in the CpG islands (2). CpG islands are characterized by a length longer than 200 bp. They possess a GC content >50% and present a ratio of observed to expected CpG dinucleotides >0.6. Moreover, CpG islands have been located in or near ~50% of human promoters (3). DNAm is catalyzed by three DNA methyltransferases (DNMTs), which have been identified in mammals: DNMT1, DNMT3A, and DNMT3B. DNMT1 maintains hemimethylated DNA patterns during DNA replication, while DNMT3A and DNMT3B establish new patterns of methylation in early embryonic development (4).

Here, we review several DNAm alterations in cancer that have been associated with carcinogenesis, apoptosis avoidance, migration, invasion, and metastasis. Several studies have found that some of these DNAm alterations may be associated with tumor clinical features such as disease risk, TNM (tumor, node and metastasis)-stage, prognosis, diagnosis, survival, and response to treatment. We also discuss several DNAm alterations in genes and some pathways that have been reported to promote tumor resistance to therapeutic agents. Additionally, we argue that the promotion or inhibition of DNAm in a non-specific way should be carefully revised because of their side effects. In contrast, more extensive studies should be further developed by considering the targeting specific alterations in DNAm or editing the epigenome by CRISPR-Cas9 technology.

## DNA Methylation Regulates Gene Expression

Genetic mechanisms and epigenetic modifications such as DNAm, histone modifications, and non-coding RNAs (including microRNAs) regulate gene expression, which is a fundamental process that maintains cellular homeostasis. Each cell type possesses its own gene expression pattern, driven by a specific epigenetic signature, which may also produce cell heritable characteristics (5).

DNAm is a covalent modification in which a methyl group is linked to the cytosine in the dinucleotides cytosine-guanine (CpG), which is often located in “CpG islands” in the gene promoters; as a consequence, DNAm can modify gene expression. The CpG island is a short sequence of DNA in which the frequency of the CpG sequence is higher than that in other regions. Hypomethylation of promoter regions allows gene expression machinery to access the promoters of target genes. Hypermethylation, on the other hand, can suppress gene expression through the modulation of the transcription machinery access to the chromatin or through the recruitment of methyl binding proteins (5). In addition to promoters, other upstream DNA regions are rich in CpG sequences, up to 2 Kb distant to CpG islands, which are named “CpG island shores.” These CpG island shores have been observed in colon and breast cancers (6, 7), or up to 700 bp in prostate cancer (8). Methylation of these CpG island shores also regulates gene expression. This epigenetic regulation was confirmed with the reactivation of downregulated genes in colon cancer, by demethylation of hypermethylated CpG island shores, using 5-aza2′-deoxycytidine (a DNA methyltransferase inhibitor) and DNA methyltransferase knockout (6).

Throughout the human lifespan, epigenetic patterns may change and constitute an important component of the aging process. Studies of human DNA methylome have revealed that one-third of 476,366 DNAm sites are affected by age. When age increases from 14 to 94 years, 60.5% of these affected DNAm sites become hypomethylated, and 39.5% become hypermethylated (9).

DNAm is a dynamic process that can also be affected by environmental factors, diet, and exercise habits, which can induce particular gene-expression signatures. For instance, the presence of short-chain fatty acids, such as butyric acid, can induce changes in DNAm patterns in normal and cancer cells; diets deficient in methyl-donor folic acid also promote dynamic changes in DNAm (5). Exercise favors hypomethylation of peroxisome proliferator-activated receptor gamma coactivator 1-alfa and delta (PGC-1α, PPAR-δ), pyruvate dehydrogenase kinase-4 (PDK4), which regulate mitochondrial function and fuel usage. mRNA upregulation of these genes during acute exercise is correlated with a transient but marked hypomethylation on each respective promoter (5). Interestingly, the change in the DNAm patterns associated with exercise is stronger among older people. The decreased DNAm associated with exercise habits among older people has been associated with cancer prevention, rewinding the “epigenetic clock” as people age (10).

## Cancer Modifies Gene Expression Through DNA Methylation

Studies of global DNA methylation have found methylation patterns or signatures that have been associated with different cancer hallmarks, such as cell proliferation, migration, invasion, and metastasis, and also with clinical features such as disease stage, prognosis, survival, and response to treatment (2). Many regulatory regions of tumor suppressor genes and oncogenes present an altered methylation pattern (6). Aberrant DNAm, mediated by the overexpression of DNMTs, affects tumor suppressor genes through their hypermethylation, leading to transcriptional silencing of these genes. On the other hand, transcriptional activation by hypomethylation is observed in proto-oncogenes. Hypomethylation may be detected in early and late stages of the tumorigenesis in several cancer types, such as lung cancer, breast cancer, prostate cancer, gastric cancer (GC), and hepatocellular cancer, among others (6).

Neoplastic transformation, carcinogenesis, and cancer progression may be led by DNAm disruption, given that epigenetic changes have been demonstrated in multiple cancers (11). Most DNAm changes in cancer occur in both CpG islands and CpG island shores, affecting the expression of tumor suppressors and oncogenes (6). For instance, it has been found that ~7,000 CpG islands are altered in the genome of human bladder cancer (12).

Analysis of methylation patterns in genomes of normal breast tissue indicates that the 5' end of highly expressed genes presents enriched sites of hypomethylation. This 5' end region includes the promoter, first intron, and first exon. In contrast, the methylome's analysis of genomes of the breast tumor cell lines (MDA-MB-231 and MCF-7) shows extensive hypomethylation in the intergenic and intragenic regions. These tumor cell lines present megabase-sized hypomethylated zones, which are associated with gene-poor regions containing tissue-specific gene clusters, fragile sites, chromosomal rearrangement breakpoints, and large genes. This suggests that hypomethylation is involved in genome instability. Interestingly, the extensively hypomethylated genes are all silenced. Also, primary breast tumors exhibit a methylation pattern that is between those of the cell lines and the normal tissue (13). It is well-documented that inactivation of tumor-suppressor genes can also be caused by deletions, point mutations, or allelic loss. Marsit et al. speculated that there might be a mutual relationship between the predisposition to promoter hypermethylation and genetic deletion in non-small cell lung carcinomas (NSCLCs). Interestingly, tumors that exhibit a high loss of heterozygosity show a reduced propensity for hypermethylation. The authors conclude that tumor suppressor gene silencing might be caused by allele loss events or epigenetic silencing events, occurring in a roughly dichotomous fashion, which would promote different molecular phenotypes in lung cancer (14).

Table 1 (15–85) summarizes genes with decreased expression in cancer as a consequence of hypermethylation of their promoter regions. The absence or reduction of the protein function associated with these genes has been implicated in the development, progression, invasion, and metastasis of many cancer types. Moreover, many hypermethylated genes included in Table 1 participate in pathways involved with cell death processes. On the other hand, Table 2 (86–120) summarizes epigenetically regulated genes by hypomethylation of their promoter regions, which have been found to be highly expressed in cancer. The expression or increased protein function associated with some of these genes has been shown to support cell proliferation, migration, and invasion of many cancer types. Many hypomethylated genes are highly expressed and participate in pathways involved in proliferation and evasion of the immune system (see Table 2). Both the hypermethylation and hypomethylation status of the regulatory regions of tumor suppressor genes and oncogenes have been tested as possible biomarkers for evaluating several parameters, such as cancer risk, diagnosis, and prognosis. Furthermore, analysis of the methylation status of certain genes may be useful for chemotherapy selection for cancer patients, and even for immunotherapy or target therapy (see Tables 1, 2).

**Table 1:** Hypermethylated promoters of genes associated with tumor suppression, prognosis, response to treatment, or as potential biomarkers.

**Cancer type**	**Hypermethylated promoter**	**Biological function associated with hypermethylation**
Breast (BC)	BRCA1, DAPK1, and RASSF1A	Associated with disease progression and poor overall survival of breast cancer patients (15)
	DACT2	Contributes to the progression of breast cancer through activation of WNT signaling pathway (16)
	ATM	Useful as a potential new biomarker for relatively young patients with breast cancer (17)
	FOXA1	Impacts parity and breastfeeding because FOXA1regulates a luminal gene expression signature in progenitor cells and represses the basal phenotype (18)
Cervical (CC)	RASSF2	Associated with shorter survival in squamous CC (19)
	RASSF1A	Increases the risk of CC (20)
	TFPI2	Important role in carcinogenesis, it correlates with cancer incidence in China (21)
	SIM1	Potential diagnostic biomarker (22)
	MEG3	Associated with worse recurrence-free and overall survival, potential plasma-based biomarker (23)
	P16INK4a	Associated with smoking habit and increased risk of cervical carcinogenesis (24)
	SALL3	HPV infection correlates with SALL3 hypermethylation and contribution to carcinogenesis (25)
	IFN-γ	Associated with tumorigenesis (26)
	KLF4	Inactivates its tumor suppressor function in cervical carcinogenesis (27)
	RAD51L3 and XRCC2	Predict late toxicity in chemoradiotherapy-treated CC patients (28)
Colorectal (CRC)	RASGRF1	Is a putative biomarker of overall survival in CRC patients (29)
	HADHB	Impacts in metastasis because HADHB reduces cancer cell migration and invasiveness (30)
	EYA4	Potential candidate screening marker in Iranian population and may improve early detection of CRC (31)
	STK33	Promising biomarker for the diagnosis, prognosis, and suitable treatment of CRC (32)
	BEND5	Promotes to cell proliferation and is a prognostic marker (33)
	FAM134B	Associated with aggressiveness and poor prognosis of colorectal adenocarcinomas (34)
	CHFR	Associated with worse overall survival in CRC patients, its loss contributes to tumorigenesis of epithelial cancers (35)
	APC 1A	Implicated in smoking-associated colorectal carcinogenesis (36)
	NDN	Promotes cell proliferation by activating the Wnt signaling pathway (37)
	hMLH1	Associated with microsatellite instability and CRC risk (38)
Gastric (GC)	EIF4E	Associated with early onset, and it is a prognostic marker for GC (39)
	GPX7	Important role in gastric tumorigenesis and progression (40)
	IGF2/DMR	Hypermethylation of IGF2/DMR in leukocyte are associated with prognosis (41)
	RAR-β	Association with histological type and clinical outcomes (42)
	TERT	A potential stool biomarker in non-invasive gastrointestinal cancer screening (43)
	MGMT	Associated with an increased risk of GC, correlation with TNM-stage (44)
	CHRDL1	Induces proliferation and metastasis by activating Akt and Erk (45)
	p16	Considered an potential early marker (46)
	miR-335	Associated with poor clinical features and prognosis (47)
	SFRP2 and DKK2	Associated with poor prognosis via the activation of Wnt/ β-catenin pathway (48)
	NDRG4	Contributes to GC risk, associated with poor prognosis (49)
	RUNX3	Associated with poor prognosis, valuable diagnostic and prognostic biomarker (50)
	ADAMTS8	Important role in the invasion and metastasis (51)
	DAL-1	Associated with GC aggressiveness, potential diagnosis biomarker (52)
Hepato-cellular	NKAPL	Predicts poor outcome in HCC patients prognostic biomarker (53)
(HCC)	HOXD10	Activates ERK signaling supporting human HCC (54)
	FHIT	Associated with live cancer risk, low FHIT expression correlates with TNM-stage, tumor size, and merging of cirrhosis of liver cancer in the Chinese population (55)
	RASSF1A	Hypermethylated RASSF1A in serum as a screen method for risk and diagnostic biomarker (56)
	HCCS1	Potential biomarker for diagnosis and prognosis of HCC patients (57)
	SOCS3	Its hypermethylation stimulates HCC development in patients with HBV (58)
	miR-142	Promotes TGF-β-mediated tumor growth and metastasis (59)
Lung (LC)	MLH1	Associated with increased risk of NSCLC (60)
	PGCP	Associated with human bronchial epithelial cells immortalization (61)
	AGTR1	Biomarker to assist the detection and diagnosis of lung squamous cell carcinoma (62)
	RASSF1A and p16INK4a	The evaluation of methylation status of both genes is a promising diagnostic method in lung cancer (63)
	RARβ	Contributes to the NSCLC tumorigenesis and may serve as a potential risk factor, diagnostic marker, and drug target of NSCLC (20)
	WIF-1	Correlates with smoking behavior, promising non-invasive biomarker using blood or pleural effusion (64)
	CDKN2A	Correlates with tobacco smoking, detected in early stages of LC carcinogenesis (14)
Ovarian (OC)	RASSF1A	Decreased RASSF1A levels in serum is a sensitive tool for diagnosis and monitoring OC (65)
	BTG1	Involved in ovarian carcinogenesis (66)
	APC	Associated with increased risk of OC, biomarker value using blood samples (67)
	miR-34a	Prognostic relevance, inverse association with grading, p53 mutation status (68)
	FANCF	Associated with the susceptibility and clinicopathologic features of epithelial OC (69)
	RUNX3 and CAMK2N1	Associated with poor clinical outcome in type II of epithelial OC after complete resection (70)
	ABCA1	Associated with poor prognosis (71)
	MEG3	Contribute to the development of epithelial OC by inability to activate p53 (72)
Pancreatic (PC)	TERT	Diagnostic value in early state I of PC, recurrence, and survival prediction (73)
	SAV1	Promotes invasion and migration, represses pancreatic cancer cell apoptosis (74)
	HOPX	Prognostic indicator of pancreatic neuroendocrine tumor (75)
	CDKN2A	Critical role in pancreatic carcinogenesis and prognostic marker value (76)
Prostate (PCa)	ST6GALNAC3 and ZNF660	Potential diagnostic and prognostic biomarkers for PCa in liquid biopsies (77)
	SOX11	Correlates with adverse clinicopathological characteristics of PCa, including higher PSA level and perineural invasion (78)
	IGF2	Relevant during early stages of tumor development, during chemotherapy or androgen deprivation (79)
	SPARC	Correlation with poorer prognosis based on specific hypermethylated CpG sites (80)
	PAQR3	Associated with perineural invasion, biomarker for detection and monitoring PCa (81)
	PCDH8	Methylation status is associated with tumor size, shape, stage, and grade, hypermethylation associated with poorer prognosis (82)
	RHCG-TCAF1	Predictive of biochemical recurrence, pathological tumor stage and pre-operative PSA (83)
	TERT	Predicts biochemical relapse (84)
	GSTP1	Marker of high risk of PCa in rebiopsy on an initially negative prostate biopsy (85)

**Table 2:** Hypomethylated promoters of genes involved in tumor progression, prognosis, or potential therapeutic targets.

**Cancer type**	**Hypomethylated promoter**	**Biological function associated with hypomethylation**
Breast (BC)	NSUN2	Associated with metastatic progression in BC, promoting cell proliferation, migration and invasion (86)
	MMP7	Distinguishes the basal-like breast cancer subtype from other triple-negative tumors (87)
	IL-10	Involved in the process of breast carcinogenesis (88)
Cervical (CC)	STK31	It could be a novel cellular target gene for the HPV16 oncogeneE7, hypomethylation biomarker for CC (89)
Colorectal (CRC)	HES1	Critical role in the progression and prognosis of CRC, associated with poor prognosis (90)
	RORA1	Correlation with stages III and IV, but not with stages I and II, biomarker for chemotherapy selection in highly advanced CRC (91)
	MUC5AC	Marker of high microsatellite instability in CRC, detects microvesicular hyperplastic polyps and sessile serrated adenoma (92, 93)
	TCF3	Prognostic value indicating recurrence in stage II and III of CRC (94)
Gastric (GC)	COX2	Associated with the intestinal type of gastric cancer (95)
	IGF2	Surrogate marker of gastric cancer risk, through IGF2 hypomethylation in blood leukocyte DNA (96)
Hepato-cellular	BORIS	Promising prognostic biomarker for the prognosis of HCC (97)
(HCC)	RNA5SP38, IL21, and SDC4P macroH2A1	Prognostic and diagnostic value associated with HCC patient survival (98)
	hsa-miR-191	Associated with poor prognosis via activation of c-MET in hepatocellular carcinoma (99)
	miR-106a miR-106a	Promotes the epithelial-to-mesenchymal transition in HCC (100) Associated with stronger invasiveness, faster cell cycle progression, increased apoptosis resistance (101)
Lung (LC)	NSD1	A tumor cell-intrinsic driver of an immune cold phenotype, associated with reduced T cell infiltration into the tumor microenvironment in LC (102)
	NY-ESO-1	Associated with poor prognosis in patients not treated with chemotherapy, prognostic marker in stage 3 NSCLCs (103)
	MUC-4	TET1 regulates MUC-4 hypomethylation, which plays crucial role in carcinogenesis and tumor invasion (104)
	AHRR and F2RL3	Reflects long-term effect of smoking on the LC risk, biomarkers for smoking exposure (105)
	ARL4C	Involved in tumorigenesis of lung squamous cell carcinoma (SqCC) (106)
	TMPRSS4	Associated with poor prognosis in SqCC, a potential therapeutic target (107)
	EYA2	Promoter factor of lung adenocarcinoma oncogenesis, altering proliferation and cell cycle distribution (108)
Ovarian (OC)	SLC6A12	Associated with poor overall survival, it is a metastasis-promoting gene in OC (109)
	CT45	Possible prognostic biomarker, immunological or therapeutic target (110)
	CA9	Correlated with a more aggressive phenotype in ovarian cancer cells (111)
	AGR2	Modulator of more aggressive cancer phenotypes (112)
	ATG4A and HIST1H2BN	Associated with poor progression-free survival and overall survival (113)
Pancreatic (PC)	SERPINB5	Diagnostic marker for pancreatic ductal adenocarcinoma from pancreatitis (114)
	MUC4	Involved in carcinogenesis, prognostic marker for pancreatic cancer (115)
	S100A4	Associated with poor differentiation, promising diagnostic marker for early detection (116)
	MET and ITGA2	Associated with poor survival, having a role in pancreatic carcinogenesis (117)
Prostate (PCa)	TFF3	Potential diagnostic biomarker for PCa (118)
	CD147	Promotes aggressive tumor progression in human PC (119)
	TFF1 and TFF3	Their overexpression in PC may serve as biomarkers (120)

## Cancer Cells Present Therapy Resistance by Changing Their DNA Patterns

One of the major reasons for the failure of cancer chemotherapy is multidrug resistance (MDR). MDR is divided into the categories of primary drug resistance, which already existed prior to chemotherapy treatment (intrinsic resistance), and acquired drug resistance, which develops during the administration of chemotherapy. This MDR is associated with the regulation and function of apoptotic pathways, intracellular pH, drug pumps, DNA damage repair ability, and drug detoxification. All of these mechanisms reduce the concentration of chemotherapeutic drugs inside the cell, and hyper- and hypomethylation of certain genes appear to play a role (121).

Multiple changes in the methylation of CpG islands and CpG island shores have been found following the acquisition of drug resistance in different cancers. Studies of global DNA methylation profiling have identified different proportions of hypermethylated genes against hypomethylated ones. Baharudin et al. performed DNAm profiling on five recurrent and 43 non-recurrent patients with colorectal cancer (CRC) with 5-fluorouracil (5-FU) treatment (122). The researchers identified 4,787 significantly differentially methylated genes in the recurrent group of CRC compared to the non-recurrent group; 3,112 genes were hypermethylated, and 1,675 genes were hypomethylated. Interestingly, many hypermethylated genes were associated with the MAPK signaling pathway, which is implicated in apoptosis regulation. Conversely, many hypomethylated genes were associated with the PI3K-AKT signaling pathway and the promotion of proliferation (122). In another study, Guo et al. compared the methylation of promoters in a genome-wide study of human lung adenocarcinoma A549 cells resistant to cisplatin (A549/CDDP) with its progenitor A549 cells. The study identified 3,617 genes with differentially methylated promoters; 2,036 were hypomethylated, and 1,581 were hypermethylated. The promoters of RAS association domain family gene 1 (RASSF1), metallothionein 1G (MT1G), and G protein-coupled receptor 56 isoform 3 (GPR56) showed significantly higher hypermethylation in A549/CDDP cells compared to the progenitor A549 cells (123). Thus, increasing evidence supports the notion that epigenetic changes are a driving force behind the acquisition of drug resistance.

## Hypermethylation of Key Genes Associated With Therapy Resistance in Cancer

Downregulation of specific genes by hypermethylation of their promoters may lead to MDR. Table 3 (124–153) shows genes whose promoters may suffer hypermethylation and have been associated with resistance to antitumoral therapy in several types of cancer. The products of some of these genes are associated with signaling pathways, such as JAK-STAT, Wnt/β-catenin, MAPK/mTOR, and FAK/Ekt. The promoter hypermethylation pattern or the downregulated gene expression are promising biomarkers for early detection of intrinsic or acquired MRD (Table 3).

**Table 3:** Hypermethylation associated with chemotherapy resistance in cancer.

**Cancer type**	**Hypermethylated promoter**	**Mechanism associated with hypermethylation and diminished expression**	**Associated resistance**
Breast (BC)	TGBI	Associated with trastuzumab resistance in HER2+ BC patients	Trastuzumab (124)
	ER-α	The formation of the ZEB1/DNA methyltransferase (DNMT)3B/histone deacetylase (HDAC)1 complex on the ER-α promoter leads to DNA hypermethylation and the silencing of ER-α. Thus, ZEB1 represses ER-α transcription.	Antiestrogen (125)
	MSH2	Biomarker for early detection of resistance, target for epigenetic therapy	Doxorubicin (126)
	MGP	Associated with chemoresistant phenotype in ER+ breast cancer cells	Doxorubicin (127)
	PSAT1	Associated with cytokine and JAK-STAT signaling, and poor clinical outcome to tamoxifen in ER positive primary tumors	Tamoxifen (128)
Cervical (CC)	SOCS	Ectopic expression of SOCS1 and SOCS3 confer radio-resistance to HeLa cells	Radiation (129)
	ZNF582	Associated with resistance to radiation and chemotherapy in HeLa cells	Radiation (130)
Colorectal (CRC)	NKX6.1	Metastasis suppressor by regulating epithelial-mesenchymal transition/outcome predictor of stage II CR patients, associated with poor prognosis	5-FU (131)
	DCR1	Silencing of DCR1 in cancer cells may promote pro-survival and pro-growth signals, predictive biomarker when a combination of irinotecan and capecitabine is used	Irinotecan (132)
	MEIS2	Possibly involved in the Wnt/β-catenin pathway to maintain CRC stemness, which leads to L-OHP resistance	Oxaliplatin (133)
	miR-26b	Tumor suppressive role of miR-26b is mediated by negatively regulating P-glycoprotein protein expression	5-FU (134)
	CCNEI, CCNDBP1, PON3, DDX43, and CHL1	Associated with the recurrence of CRC and 5-azadC-mediated restoration of 5-FU sensitivity is mediated at least in part by MAPK signaling pathway.	5-FU (122)
Gastric (GC)	TFAP2E	High expression of miR 106a 5p and miR 421 regulate the chemoresistance induced by TFAP2E methylation	5-FU (135)
	TFAP2E	The lack of response to fluorouracil-based chemotherapy is associated with TFAP2E hypermethylation, indicating that it might be a potential predictor of treatment response in patients with GC	5-FU (136)
Hepato-cellular (HCC)	CSF3R KCNQ1	Associated with poor prognosis, higher recurrence rates, indicative of non-CDDP regimens in hepatoblastoma patients Remarkable inhibitory roles on tumor metastasis *in vitro* and *in vivo*	Cisplatin (137) Cisplatin (138)
Lung (LC)	PDE3A	Inhibitor of DNA synthesis and cell viability in cancer cells/PD3A re-expression improves overall survival in adenocarcinoma patients.	Cisplatin (139)
	LRP12	Associated with shorter survival, marker for carboplatin resistance	Carboplatin (140)
	miR-483-3p	Because miR-483-3p directly targets integrin β3, and represses downstream FAK/Erk signaling pathway, its absence promotes acquired EGFR TKI resistance in EGFR-mutant NSCLC	Gefitinib (141)
	GPR56, MT1G, and RASSF1	Potential methylation markers associated with acquired methylation in multidrug resistance of lung adenocarcinoma	Cisplatin (123)
Ovarian (OC)	UCHL1	Knockdown of UCHL1 reduces cell apoptosis contributing to cisplatin resistance in OC cells	Cisplatin (142)
	OXCT1	Silencing of OXCT1 is associated with cisplatin resistance	Cisplatin (143)
	BRCA1	Loss of promoter hypermethylation restore BRCA1 function in recurrent disease	Cisplatin (144)
	miR-199a-3p	Favors migratory, invasive and tumorigenic capabilities, and cisplatin resistance	Cisplatin (145)
	hMSH2	Associated with platinum resistance, poor prognosis value	Platinum (146)
	RASSF1A	Associated with multidrug resistance	Platinum and Placlitaxel (121)
	NAGA	NAGA acts as a cisplatin sensitizer	Cisplatin (147)
	TRIB2	Downregulation of TRIB2 contributes to platin-resistance, promising prognostic and predictive marker	Cisplatin (148)
	miR-490-3p	miR-490-3p enhances CDDP sensitivity of OC cells through downregulating ABCC2 expression.	Cisplatin (149)
Pancreatic (PC)	BNIP3 miR-132	Associated with chemoresistance in pancreatic ductal adenocarcinoma cell lines Promotes TGF-β-driven progression of pancreatic cancer	Gemcitabine (150) Dexamethasone (151)
Prostate (PCa)	miR-34a	Diminished miR-34a expression enhances chemoresistance, allowing upregulation of ATG4B-induced autophagy through AMPK/mTOR pathway	Dox, Topo (152)
	miR-205 and miR-31	Associated with apoptosis resistance in advanced PCa, the antiapoptotic genes BCL2L2 (encoding Bcl-w) and E2F6 have been identified as the targets of miR-205 and miR-31, respectively.	Docetaxel and Cisplatin (153)

The transcriptional silencing mediated by hypermethylation can be used as a therapeutic strategy to diminish the expression of genes associated with drug resistance. For instance, it has been shown that the transmembrane ectoenzyme CD13 endows GC patients with insensitivity to CDDP and that expression of this molecule predicts a poor prognosis in CDDP-treated GC patients. CD13 functions upstream of the epithelial membrane protein 3 (EMP3) to induce its expression. The optimal phosphorylation of PI3K is facilitated by EMP3 upregulation. Phosphorylated PI3K activates the PI3K/AKT/NF-κB pathway suppressing autophagy and epithelial-mesenchymal transition (EMT) and overcoming CDDP resistance in GC cells. Ubenimex, a CD13 inhibitor, induces transcriptional silencing of EMP3 that is mediated by hypermethylation. Therefore, to overcome CDDP resistance in GC cells, ubenimex epigenetically inhibits the activation of the CD13/EMP3/PI3K/AKT/NF-κB pathway (154). Additionally, the NFκB pathway participates in the acquisition of resistance to tyrosine kinase inhibitor (TKI) treatment in lung cancer. Although it is a rare event, methylated cytosine may be converted to thymine by deamination. As a result, the methylated CG sequence could be converted into the TG sequence. Treatment with EGFR TKIs leads to activation of the NFκB pathway and also induces the activation-induced cytidine deaminase (AICDA) expression. AICDA deaminates the 5-methylcytosine, resulting in thymine to generate a T790M mutation. Hence, this is also a methylation-associated mechanism behind the acquisition of a mutation that provides resistance to TKI treatment in lung cancer (155).

DNAm-induced silencing of tumor suppressors is common in cancer. The hypermethylation can be reverted using the FDA-approved DNMT inhibitor 5-aza-2'-deoxycytidine, also named 5-azacytidine or decitabine. 5-Azacytidine has proven to be effective in the treatment of hematological neoplasms. However, its antitumor effect varies in solid tumors (156). The inhibition of methylation has presented good results in GC; Zhang et al. reported that growth arrest-specific transcript 5 (GAS5), which is a tumor suppressor lncRNA, is downregulated in GC. Adriamycin (ADM)-resistant cells (SGC-7901/ADM) have significantly higher levels of hypermethylation in the GAS5 promoter than GC SGC-7901 cells. The authors enforced GAS5 expression, provoking a significant reduction in tumor growth rate and apoptosis after Adriamycin treatment (157). Additionally, Wu et al. have shown that hypermethylation of miR-129-5p CpG island promotes miR-129-5p downregulation, favoring chemoresistance in GC cells. In the GC MDR cell line (SGC7901/VCR), the expression of miR-129-5p was restored through the use of 5-azacytidine, which reduced the chemo-resistance to 5-FU, vincristine, and cisplatin in this cell line. When the authors downregulated miR-129-5p, the chemoresistance was recovered. Furthermore, three members of ABC transporters (ABCG1, ABCC5, and ABCB1), which are associated with MDR, are direct targets of miR-129-5p regulation (158). In contrast, the demethylation of regulatory regions can induce chemoresistance in cervical cancer. Sensitivity to DNA topoisomerase I inhibitors in cancer therapy can be affected by DNA hypermethylation of the Werner (WRN) gene that reduces WRN expression. The WRN gene codes for a DNA helicase that contributes to genomic stability. Masuda et al. reported that cervical cancer-derived cell lines and primary cervical cancer that presented decreased WRN expression due to DNA hypermethylation showed high sensitivity to the topoisomerase I inhibitor (CPT-11). After treatment with 5-azacytidine, the tumor cells became resistant to CPT-11. To confirm this result, they transfected with a siRNA against WRN in tumor cells. These cells increased the sensitivity to CPT-11 (159). Therefore, treatment with demethylating drugs may have unforeseen and opposing results in cancer patients.

Silencing mechanisms to prevent the expression of tumor suppressor genes may also be induced by hypermethylation in cancer. For instance, potassium (K+) channels are dysregulated in different tumors and contribute significantly to the malignant phenotypes, such as chemoresistance, proliferation, and migration. KCNQ1 (potassium channel) can interact with β-catenin to affect its subcellular distribution. The interaction of KCNQ1 with β-catenin reduces Wnt/β-catenin signaling, which consequently blocks the expression of its downstream targets, such as MMP7, CCND1, and c-Myc. As a result, proliferation and cell migration are inhibited. DNA hypermethylation of KCNQ1 promoter has been shown to downregulate KCNQ1 expression in hepatocellular carcinoma (HCC). Downregulation of KCNQ1 is found in HCC cell lines and tissues and is associated with a poor prognosis (138). Additionally, the KCNQ1 Opposite Strand/Antisense Transcript 1 (KCNQ1OT1) gene is a lncRNA, which has been reported to be highly expressed in colorectal and lung cancers. High KCNQ1OT1 expression is correlated with malignant phenotypes in lung cancer. The transfection of si-KCNQ1OT1 can effectively knock down the expression of KCNQ1OT1, increasing KCNQ1 levels and, thus, inhibiting the malignancy and chemoresistance of lung cancer cells to paclitaxel (160). Accordingly, treatments that focus on recovering KCNQ1 expression must consider both the hypermethylation of regulatory regions and the expression of lncRNA. Another example of multiple mechanisms for silencing gene expression is the downregulation of BCL2 interacting protein 3 (BNIP3), which is a proapoptotic member of the BCL-2 family that induces necrotic-like cell death. Loss of BNIP3 expression in pancreatic cancer is correlated with methylation of the BNIP3 promoter. Mahon et al. showed an association between the decreased expression of BNIP3 and chemoresistance to gemcitabine in pancreatic ductal adenocarcinoma (PDAC) cell lines. Besides promoter hypermethylation, S100A4 overexpression, which belongs to the S100 calcium-binding protein family, represents an alternative mechanism for inhibiting BNIP3 function in PDAC. S100A4 knockdown, mediated by RNA interference, upregulated the expression of BNIP3 in PDAC cell lines that have an unmethylated BNIP3 promoter, which led to an increased sensitivity to gemcitabine in PDAC cell lines (150). Consequently, it is important to keep in mind that hypermethylation is one of several mechanisms that inhibits tumor suppressor genes, and anti-cancer treatments must consider this fact.

## Hypomethylation of Key Genes Is Associated With Therapy Resistance in Cancer

Hypomethylation of promoters for a certain type of gene may also function as tumor mechanisms for acquiring resistance to drug therapy. Table 4 (161–181) shows some genes whose promoters are hypomethylated in several types of cancer. Hypomethylation of the promoters of these genes leads to their upregulation. The product of some of these genes supports mechanisms involved in MDR, proliferation, the repression of apoptotic signaling, mitochondrial function, and DNA repair. For instance, Luzhna et al. found that diminished radiation responsiveness was correlated with significant global DNA hypomethylation in radiation-resistant cells (MCF-7/DOX). This radiation resistance can be reversed by an epigenetic treatment, which is the use of SAM, a methyl donor. The radiation sensitivity in MCF-7/DOX cells was promoted through use of the SAM-mediated reversal of DNA methylation. However, the researchers found that SAM should be carefully used because the SAM application decreased responsiveness to radiation on MCF-7 cells that were originally radiation-sensitive and highly methylated. Remarkably, the authors concluded that a fine balance of DNA methylation is needed to ensure proper drug and radiation responsiveness (182).

**Table 4:** Hypomethylation associated with chemotherapy resistance in cancer.

**Cancer type**	**Hypomethylated promoter**	**Mechanism associated with hypomethylation and increased expression**	**Associated resistance**
Breast (BC)	ID4	Potential biomarker in distinguishing acquired tamoxifen-refractory BC	Tamoxifen (161)
	ERp29/ MGMT	ERp29 expression in the triple negative MDA-MB-231 breast cancer cells significantly increases cell survival against ionizing radiation, by downregulating DNA methyltransferase 1, ERp29 promotes promoter's hypomethylation of the DNA repair gene (MGMT)	Radiation (162)
	ETS-1	Inhibitor of miR-320a expression, downregulation of miR-320a triggers TRPC5 and NFATC3 overexpression, which are essential for BC chemoresistance	Adriamycin and paclitaxel (163)
	miR-663	Overexpression of hypomethylated miR-663 induces chemoresistance in breast cancer cells by down-regulating HSPG2.	Cyclophosphamide and docetaxel (164)
	MDR1, GSTpi, MGMT, and Upa	Hypomethylation of the promoter regions of the MDR1, GSTpi, MGMT, and Upa genes is associated with acquirement of doxorubicin resistance of MCF-7 cells	Doxorubicin (165)
Colorectal (CRC)	NME2	Enhancer of growth abilities and reduced apoptosis in HCT-8 cells	5-FU (166)
	CDO1	CDO1 hypomethylation in stage III colon cancer with postoperative chemotherapy exhibits worst prognosis than CDO1 hypermethylation. In some CRC cell lines, forced expression of CDO1 gene increases mitochondrial membrane potential accompanied by chemoresistance and/or tolerance under hypoxia.	Adjuvant (167)
	Nrf2	TET-dependent demethylation of the Nrf2 promoter upregulates Nrf2 and HO-1 expression, which induces cellular protection mechanisms, leading to 5-FU resistance in CRC cells	5-FU (168)
Gastric (GC)	ASCL2	Enhanced ASCL2 expression increases cell growth and promotes resistance to 5-FU in GC cells, a useful prognostic marker for GC patients	5-FU (169)
	MDR1	Overexpression of DCTPP1 decreases the concentration of intracellular 5-methyl-dCTP, which results in promoter hypomethylation and hyper-expression of MDR1	5-FU (170)
	GTSE1	GTSE1 expression represses apoptotic signaling and confers cisplatin resistance in gastric cancer cells.	Cisplatin (171)
Hepato-cellular (HCC)	PD-L1/DNMT1 axis	Highly DNMT1 upregulation positively correlates with PD-L1 overexpression in sorafenib-resistant HCC cells, where PD-L1 induced DNMT1-dependent DNA hypomethylation	Sorafenib (172)
	MDR1	MDR1 promoter hypomethylation might be regulated by the riboregulatory H19, inducing the P-glycoprotein expression through the upregulation of its gene MDR1 in liver cancer cells	Doxorubicin (173)
Lung (LC)	TDRD9	Associated with aberrant mitosis and abnormal-shaped nuclei, protects from replicative stress increasing drug resistance	Aphydicolin (174)
Ovarian (OC)	SERPINE1	Associated with EMT process and carboplatin resistance in A2780cp cells	Carboplatin (175)
	TMEM88	Functions as an inhibitor of Wnt signaling contributing to the platinum resistance	Platinum (176)
	BRCA1/SIRT1/EGFR axis	Cisplatin-resistant ovarian cancers increase BRCA1, SIRT1, and EGFR levels compared with those in cisplatin-sensitive ovarian cancers. Decreased nicotinamide adenine dinucleotide (NAD)-mediated SIRT1 activity, decreased EGFR levels, significantly elevated SIRT1 levels, and BRCA1 activation are associated with hypomethylation in the BRCA1 promoter	Cisplatin (177)
	HERV	HERV-K hypomethylation is associated with a poor prognosis and platinum resistance in ovarian clear cell carcinoma (OCCC), promising biomarker for predicting OCCC treatment response and prognosis.	Platinum (178)
	MAL	Highly expressed MAL gene in serous ovarian cancers from short-term survivors (<3 years) and treated with platinum-based therapy. MAL methylation status is a potential target for enhancing sensitivity to platinum-based drugs in epithelial ovarian cancer	Platinum (179)
Prostate (PCa)	miR-27a-5p	miR-27a-5p promoter becomes hypomethylated during PCa progression, miR-27a-5p upregulation decreases EGFR/Akt1/mTOR signaling	Castration (180)
	CD117 and ABCG2	Prostate cancer cell line 22RV1 expresses high surface levels of both CD117 and ABCG2 (CD117+ABCG2+ cells). This subpopulation shows hypomethylation in ABCG2 promoter and also overexpresses stem cells markers such as Nanog, Oct4, Sox2, Nestin, and CD133	Cisplatin, paclitaxel, adriamycin, and methotrexate (181)

Activation of drug-resistance-associated genes, besides the hypomethylation of their promoters, can be caused by *de novo* gene fusions. In the case of breast cancer, BRCA1-deficient tumors are extremely sensitive to DNA-damaging drugs and poly(ADP-ribose) polymerase (PARP) inhibitors. However, BRCA1 protein was detected in 31 of 42 drug-resistant cases, despite presenting a hypermethylated promoter. BRCA1-intragenic deletions and the loss of BRCA1 promoter hypermethylation have been shown to occur, and *de novo* gene fusions take place, where BRCA1 expression can be under the transcriptional control of a heterologous promoter (183).

Therefore, targeting methylation should be carefully evaluated because most compounds that promote or inhibit this process are not gene-specific, which may lead to undesirable effects.

## Regulation of Wnt Canonical and NON-Canonical Pathways by DNA Methylation that Supports Cancer Development and Therapy Resistance

In this section, we include potential mechanisms by which differentially methylated genes take part in the development of cancer, by integrating protein interactions and pathways regulated by methylation (11). Tumors present a dysregulated pattern of methylation in genes that impact in pathways like the Wnt canonical pathway and PI3K/AKT/mTOR (e.g., DKK, SFRPs, WIF1, DVL, APC, PTEN, SALL2, and IGFBP-3), which support resistance to specific inhibitors as well as conventional chemotherapeutic agents.

**Figure 1:**
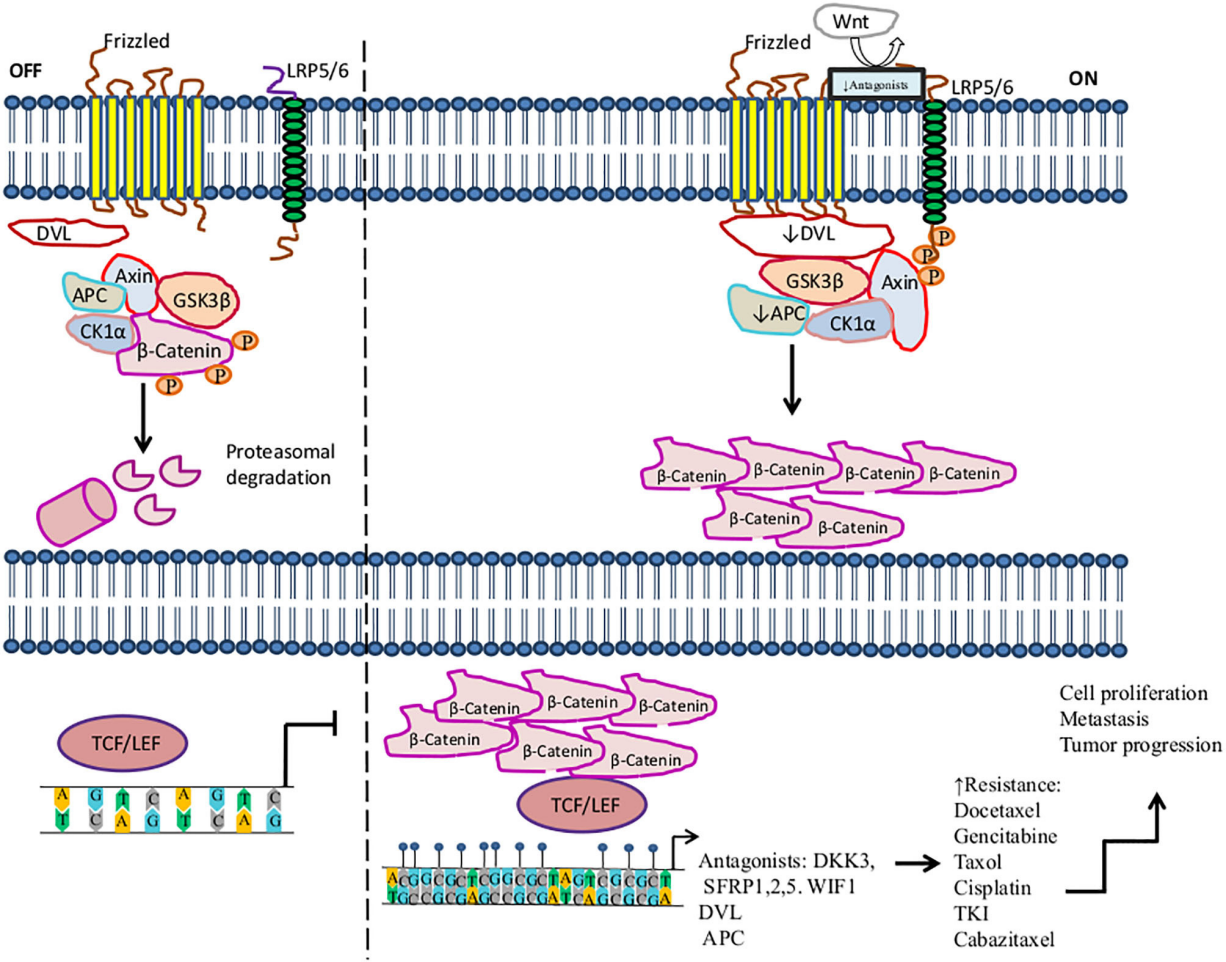
Activation of the Wnt/β-catenin signaling pathway in the resistance to therapy in cancer by methylation. The canonical Wnt/ß-catenin pathway is activated by the binding of Wnt to the Frizzled receptor (Fzd). Then LRP is phosphorylated by casein kinase 1 (CK1α) and glycogen synthase kinase 3 beta (GSK3β) associated with tumor suppressor adenomatous polyposis coli (APC). LRP phosphorylation promotes the recruitment of disheveled proteins (DVL) to the plasma membrane, where they are polymerized and activated. DVL complex interacts with Axin, which inhibits the degradation of β-catenin and leads to its accumulation in the cytoplasm and translocation into the nucleus, where β-catenin promotes the activation of LEF/TCF transcription factors inducing the transcription of several genes. In the absence of Wnt, β-catenin is a target of the destruction complex conformed by Axin, CK1α, APC, and GSK-3β. CK1α and GSK-3β phosphorylate β-catenin, promoting its ubiquitination by the β-TrCP ubiquitin ligase and degradation through the proteasome. On the other hand, this figure shows some aberrantly methylated key genes that increase resistance to therapeutic agents and the dysregulation of the Wnt/β-catenin signaling pathway. These genes play an antagonist role in the Wnt pathway, for instance, Dickkopf-related protein (DKK3), secreted frizzled-related proteins (SFRP1, SFRP2), and WNT inhibitory factor-1 (WIF1), which are tumor suppressor genes and inhibit the signaling of Wnt to bind LRP5/6 receptors. 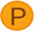, phosphorylation; ↑, increase; ↓, decrease,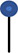, methylation.

The Wnt/beta-catenin signaling pathway plays important roles in carcinogenesis and therapy resistance. Wnt is a large family of secreted lipoproteins that can join to receptors and co-receptors at the cell surface and activate a complex signaling network. This pathway participates in a wide range of physiological cellular processes like embryonic development, tissue homeostasis, tissue regeneration, cell polarity, cell proliferation, cell migration, and apoptosis (184, 185). The Wnt signaling pathway may be activated by the Wnt/ß-catenin pathway (also known as the canonical pathway (Figure 1) (185, 186) or non-canonical Wnt signaling. Non-canonical Wnt signaling, which is independent of β-catenin, is activated by the pathways Wnt/planar cell polarity and Wnt/Ca^2+^ (187, 188).

Methylation of genes involved in the Wnt pathway plays a crucial role in regulating the development and progression of tumors, as well as metastasis, diagnosis, and treatment. Several tumors, such as lung, breast, prostate, colon, gastric, and ovarian cancers, among others, exhibit a pattern of deregulated methylation in this pathway (184, 189, 190). For instance, Dickkopf-related protein (DKK3), secreted frizzled-related protein 1 (SFRP1), SFRP2, and Wnt inhibitory factor-1 (WIF1), which are tumor suppressor genes, prevent LRP5/6 receptors from interacting with their ligands, consequently inhibiting the signaling of the Wnt pathway (191, 192). In the context of methylation, it has been said that several tumors show the downregulation of DKK3, SFRP1, SFRP2, and WIF1 by hypermethylation in their promoters (193). The hypermethylation of DKK3 has been associated with docetaxel (DTX) resistance in the lung cancer H1299/DTX cell line. Moreover, treatment with 5-azacytidine on the H1299/DTX cell line upregulates DKK3 expression at both the mRNA and protein levels, which inhibits colony formation and induces apoptosis due to recovered sensitivity to DTX. Additionally, P-glycoprotein is a drug efflux pump associated with MDR, encoded by the MDR-1 gene (MDR-1). MDR-1 overexpression is associated with DTX resistance in lung cancer. Restored expression of DKK3 leads to the downregulation of MDR-1 and P-glycoprotein, thus increasing sensitivity to DTX. This is a mechanism of regulation in lung cancer therapy. Therefore, DKK3 may be a therapeutic target that may help tumor cells recover sensitivity to DTX (194). Another study found that the decreased expression of DKK3 is associated with hypermethylation in pancreatic cancer biopsies in comparison to non-tumor tissue. In this study, DKK3 expression was not detected in three pancreatic cancer cell lines (Aspc-1, Bxpc-3, and CFPAC-1). DKK3 overexpression by DKK3 transfection in the Bxpc-3 pancreatic cell line promotes the inhibition of β-catenin translocation to the nucleus, as well as its transcriptional role under conditions of hypoxia or normoxia. Furthermore, DKK3 repressed the EMT and migration of Bxpc-3 cells, mediated by the inhibition of β-catenin. These effects improved the response to gemcitabine in Bxpc-3 tumor cells, suggesting that DKK3 may be a potential target for therapy (195).

In advanced stages of lung cancer, treatment based on taxanes is one treatment option, such as paclitaxel and DTX; however, resistance to therapy is presented in some patients (196). Ren et al. showed that hypermethylated SFRP1 regulates the chemotherapy resistance of taxanes and DTX in A-549 and SPC-A1 lung adenocarcinomas cell lines. The resistance was mediated by Wnt pathway activation because SFRP1 reduces β-catenin stability, leading to cell death, whereas SFRP2 promotes β-catenin accumulation, inducing resistance to apoptosis. Moreover, 5-azacytidine treatment restored the SFRP1 expression level, inducing the inhibition of the Wnt pathway and promoting drug sensitivity in resistant cell lines. Thus, the overexpression of SFRP1 can improve patients' responses to taxanes and DTX therapies (196). Zhu et al. also found that SFRP1 and SFRP5 were hypermethylated in NSCLC. Furthermore, the hypermethylation of SFRP5 predicted a poor response to TKI therapy; hence, SFRP5 methylation could be associated with TKI resistance (197). Additionally, higher levels of the hypermethylation of SFRP1, SFRP2, and WIF1 genes were found in colon cancer compared to non-tumoral tissues. Thus, the hypermethylation of one or both SFRP1and SFRP2 genes is a promising prognostic marker for predicting survival in patients who receive post-operative chemotherapy (198–200). Su et al. found that SFRP5 was hypermethylated in 44.4% of ovarian cancer tissues, as well as in SKOV3 and A2780 tumor cell lines. The SFRP5 low expression promotes EMT, tumor growth, invasion, tumor progression, and cisplatin resistance. Restored expression of SFRP5 reduces Wnt non-canonical signaling, promoting sensitivity to cisplatin in a mouse model of ovarian cancer (201).

Several genes whose protein products participate in the Wnt transduction signaling cascade are regulated by hypermethylation. Hence, the deregulation of this process during carcinogenesis may contribute to drug resistance. For instance, hypermethylation of DVL in prostate cancer has been suggested to favor resistance to cabazitaxel in DU145 cells. Reactivation of DVL by 5-azacytidine treatment in DU145 10DRCR cells restores sensitivity to cabazitaxel in this prostate tumor cell line (202). Hypermethylation of adenoma polyposis coli (APC), a tumor suppressor gene, inhibits the Wnt pathway, promoting tumorigenesis and tumor progression in CRC (203, 204). In breast cancer, Matuschek et al. reported that hypermethylated APC promotes tumor aggressiveness in circulating tumor cells. Additionally, 70% of breast cancer tissues presented hypermethylation in the APC gene (205). By the same token, loss of APC inactivates the repair of double-stranded breaks mediated by ATM, Chk1, and Chk2, which induces doxorubicin resistance (205, 206). Thus, we suggest that the methylation status of several key genes involved in the Wnt/canonical signaling pathway may be used as predictive markers of tumor progression and therapy response.

## Regulation of the PI3K/PTEN/AKT/mTOR Signaling Pathway by DNA Methylation Supports Cancer Development and Therapy Resistance

**Figure 2:**
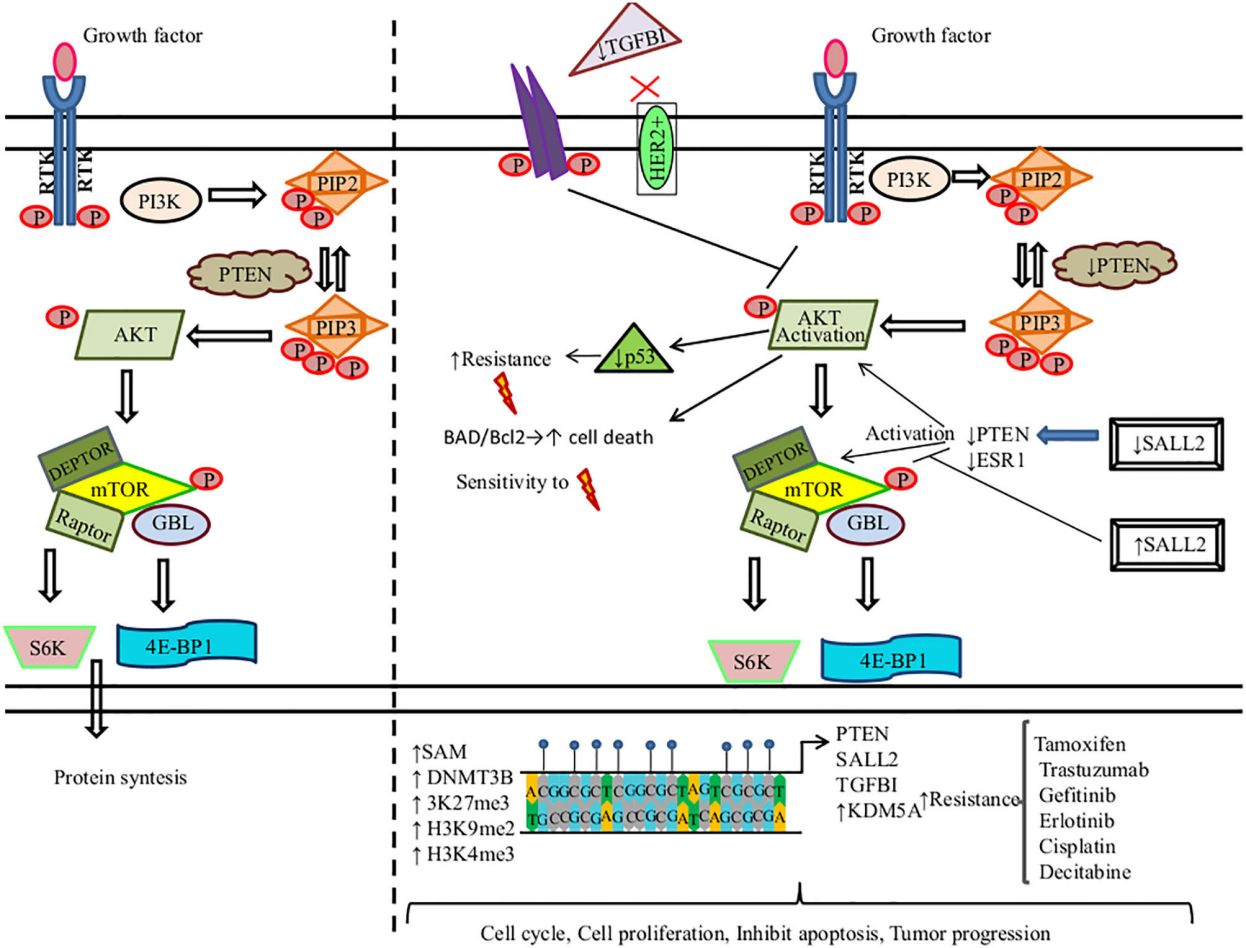
DNA methylation regulates the PI3K/PTEN/AKT/mTOR signaling pathway in the resistance to therapy in cancer. **(Left)** PI3K induces the phosphorylation and activation of AKT/mTOR. This transduction signal begins with the activation of the membrane tyrosine kinase receptors (RTKs) or G-protein-coupled receptors, which promotes the change of phosphatidylinositol (4,5)-bisphosphate (PIP2) in phosphatidylinositol (3-5)-trisphosphate (PIP3). The activation of PI3K (phosphoinositide-3-kinase) is regulated by the phosphatase and tensin homolog (PTEN) by dephosphorylating PIP3 into PIP2. **(Right)** We show the aberrant methylation of the PTEN, Spalt-like transcription factor 2 (SALL2), transforming growth factor beta-induced protein (TGFB1), and Lysine (K)-specific demethylase 5A (KDM5A) genes through the high expression of methyltransferase (DNMT3B), s-adenosylmethionine (SAM), H3K27me3, H3K9me2, and H3K4me3, promoting a continued activation of the PI3K/AKT/mTOR signaling pathway associated with resistance therapy in solid tumors. 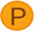, phosphorylation; ↑, increase; ↓, decrease; 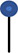, methylation, 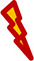, radiotherapy.

AKT is also recognized as protein kinase B (Serine/Threonine Kinase 1, or Protein Kinase B), which participates in several processes such as cell metabolism, cell proliferation, angiogenesis, apoptosis, motility, and cell survival (207). Aberrant DNAm of key genes in PI3K/PTEN/AKT/mTOR signaling pathway promotes therapy resistance in solid tumors (Figure 2).

Tamoxifen (TAM) is the first line of therapy for the treatment of estrogen-receptor-positive breast cancer. This type of breast cancer develops TAM resistance, promoting tumor relapse (208). Phuong et al. found that the MCF-7 breast cancer cell line showed TAM resistance (TAMR/MCF-7). This resistance is mediated by a high expression of DNMT1. DNMT1 along with SAM induce the hypermethylation of PTEN in amplicon A and amplicon B sites, leading to its downregulation and the constitutive activation and phosphorylation of PI3K/AKT. 5-Azacytidine treatment inhibits DNMT1 in TAMR/MCF-7 cells, restoring PTEN expression, suppressing cell proliferation, and promoting cell death by apoptosis (209). Spalt-like transcription factor 2 (SALL2) functions as a tumor suppressor, which regulates the AKT/mTOR pathway (Figure 2). Hypermethylated SALL2 is found in the TAM-resistant ER+ TAMR/MCF-7 breast cancer cell line, which leads to SALL2 downregulation. SALL2 decreased expression promotes decreased expression levels of estrogen receptor-alpha (ERα) and PTEN, which causes the continued activation of AKT/mTOR. In addition, hypomethylation of SALL2 increases its expression, leading to the upregulation of ER and PTEN and the further inhibition of the AKT/mTOR signaling pathway, which consequently leads to TAM sensitivity (210).

Among breast cancers, 15–20% are human epidermal growth factor receptor 2 positive (HER2+) and may develop resistance to trastuzumab. Palomeras et al. reported that primary breast tumors that developed trastuzumab resistance present a loss of expression of transforming growth factor beta-induced protein (TGFBI) by hypermethylation. TGFBI inhibits the HER2 receptor and AKT (124). Abnormal activation of PI3K/AKT is common in breast cancer. Among the most frequent causes is constitutive signaling through mutational activation of phosphatidylinositol-4,5-bisphosphate 3-kinase catalytic subunit alpha (PIK3CA), which is mutated in 45% of luminal breast cancers. Thus, a promising therapeutic strategy is to develop PI3K/AKT inhibitors. KDM5A lysine demethylase can remove tri- and dimethyl marks on histone H3 (H3K4me3), leading to tumor progression and drug tolerance. KDM5A is a target of AKT and, together, they regulate certain cell-cycle genes. AKT phosphorylates KDM5A, thus promoting the subcellular localization of KDM5A from the chromatin-bound regions and nucleus to the cytoplasm. As a result, KDM5A is rendered unable to demethylate H3K4me2/3. PI3K/AKT inhibition decreases KDM5A phosphorylation, promoting the low expression of cell-cycle promoting genes. Additionally, KDM5A regulates resistance to PI3K/AKT inhibitors (211).

On the other hand, some mutated EGFR lung cancers induce resistance to EGFR-TKIs (gefitinib and erlotinib). Two gefitinib-resistant cell lines (GEF1-1 and GEF2-1) derived from the PC-9 cell line were treated with 5-azacytidine. This treatment restored PTEN expression and promoted sensitivity to gefitinib and erlotinib in GEF1-1 and GEF2-1 cell lines. Nonetheless, the parental cell line (PC-9 cells) did not show this sensitivity due to the hypermethylation of PTEN and hyperactivation of AKT (212). Furthermore, the hypermethylation of insulin-like growth factor-binding protein-3 (IGFBP-3) promotes cisplatin resistance in lung cancer. Downregulation of IGFBP-3 induces PI3K/AKT activation by specific de-repression of insulin-like growth factor-I receptor (IGFIR) signaling (213).

Methylation of PTEN promoter is an alternative mechanism to PTEN downregulation that induces drug resistance. For instance, lung cancer cells develop radioresistance due to hypermethylated PTEN that induces low expression of pAKT and downregulates p53 expression (214). In similar research, Pappas et al. showed that restoring PTEN expression in the human lung cancer cell line H1299 by the use of the adenovirus expression vector (Ad-PTEN) increases sensitivity to ionizing therapy. Of note, PTEN promoter is methylated in H1299 cells. The phosphorylation of BAD, a proapoptotic molecule regulated by AKT, inhibits its binding to Bcl-2, leading to apoptosis. Thus, restoring PTEN induces lower levels of phosphorylated AKT and BAD, which sensitizes to apoptosis. Also, Ad-PTEN regulates the DNA repair of double-strand breaks, mediated by the activation of H2AX (215).

Differential DNA methylation profiles are found in prostate cancer samples. These tumors show hypermethylated PTEN and hemi- and homozygous PTEN loss. The latter has been associated with poor prognosis, recurrence, and tumor progression (216).

Qian et al. found that DNMTs were highly expressed in nasopharyngeal carcinoma resistant cells; consequently, PTEN and PPP2R2B promoter hypermethylation is induced. DNMT upregulation activates two important signaling pathways, PI3K/mTOR and PDK1/MYC, favoring survival, proliferation, and resistance to the BEZ235 inhibitor (217). Treatment with BEZ235 and the inhibition of DNMT expression with 5-azacytidine induces drug sensitivity in resistant tumor cells. Furthermore, 5-azacytidine dephosphorylates the AKT, GSK3β, MYC, P70, and 4EBP-1 proteins involved in the AKT/mTOR and PDK1/MYC pathways. The combination of decitabine and BEZ235 upregulates PTEN protein expression, inhibiting cell growth. Hence, new combinations of chemotherapeutic agents with inhibitors against components of the PI3K/AKT/mTOR signaling pathway should be tested to increase tumor chemotherapy sensitivity.

## Conclusions and Future Directions

Several studies have shown that modifications in DNAm patterns may support cancer development, invasion, and metastasis. Global methylation analyses suggest that CpG islands tend to be a highly altered methylation status that depends on the cancer type (122, 123). Importantly, several studies have reported that DNAm patterns of drug-treated tumor cells can change and support the acquisition of resistance to treatments, such as radiotherapy and chemotherapy. Some of these DNAm changes have been proposed as promising biomarkers whose presence would be indicative that therapy must be replaced (Tables 3, 4).

Additionally, the study of the DNAm patterns and their involvement in the regulation of several signaling pathways in cancer has provided significant insight into the molecular mechanisms underlying the development of cancer. For instance, dysregulation of Wnt canonical and PI3K/AKT/mTOR signaling pathways, caused by an altered methylation status in a variety of genes, has been associated with resistance to current treatments (taxanes, DTX, cisplatin, TKI, etc.) in many types of cancer.

Some studies have tried to change drug-resistance-associated DNAm patterns using SAM and DNMTs to increase the methylation grade or TET-dependent demethylation to diminish DNAm. Although they have changed the DNAm pattern of the target gene, these treatments also modify the DNAm patterns of other genes, causing undesirable secondary effects. For this reason, a fine balance of DNAm is needed to ensure proper drug responsiveness.

Recent advances in applied genetic engineering and genome editing may provide new tools for targeting methylation status in cancer patients. In particular, the clustered regularly interspaced short palindromic repeats/associated protein 9 (CRISPR/Cas9) system allows for the addition or removal of DNA from the genome in a specific manner. Genetic engineering has produced a new version of Cas9 (dCas9), in which the activity of endonuclease has been removed but in which the DNA binding activity is maintained. dCas9 can also be linked to DNMT3A or ten-eleven translocation-1 (TET1) enzymes, generating the systems dCas9-DNMT3A or dCas9-TET1, respectively (218, 219). These systems can induce the methylation and demethylation of target genes in a specific-sequence manner, which makes them promising tools in the fight against cancer or the acquisition of therapy resistance.

## Author Contributions

SR-G and AC-R designed the study. SR-G, HP-G, and AC-R wrote the manuscript and integrated the tables. AC-R designed and elaborated the figures. All authors contributed to the critical revision of the manuscript, read, and approved the submitted version.

## Conflict of Interest

The authors declare that the research was conducted in the absence of any commercial or financial relationships that could be construed as a potential conflict of interest.
